# Chinese version of the Constant-Murley questionnaire for shoulder pain and disability: a reliability and validation study

**DOI:** 10.1186/s12955-017-0752-3

**Published:** 2017-09-18

**Authors:** Min Yao, Long Yang, Zuo-yuan Cao, Shao-dan Cheng, Shuang-lin Tian, Yue-li Sun, Jing Wang, Bao-ping Xu, Xiao-chun Hu, Yong-jun Wang, Ying Zhang, Xue-jun Cui

**Affiliations:** 1 Institute of Spine Disease, Longhua Hospital Affiliated to Shanghai University of Traditional Chinese Medicine, No. 725, Wanping south road, Shanghai, 200032 China; 2 Key Laboratory of Theory and Therapy of Muscles and Bones, Ministry of Education (Shanghai University of Traditional Chinese Medicine), No. 725, Wanping south road, Shanghai, 200032 China; 3 Rheumatism Department, Longhua Hospital Affiliated to Shanghai University of Traditional Chinese Medicine, No. 725, Wanping south road, Shanghai, 200032 China; 4 Department of Neck Shoulder Waist and Leg Pain, Shanghai Guanghua integrative medicine Hospital, No. 540, Xinhua road, Shanghai, 200052 China; 5 Shanghai Hospital of Traditional Chinese Medicine, Shanghai University of Traditional Chinese Medicine, No. 274, Zhijiang road, Shanghai, 200071 China; 6 Department of Orthopedics, Shanghai Changzheng Hospital, No. 415, Fengyang road, Shanghai, 200003 China; 7 Department of Orthopaedics, Longhua Hospital, Shanghai University of Traditional Chinese Medicine, No.1200 Cai Lun Road, Shanghai, 201203 China

**Keywords:** Construct validity, Internal consistency, Shoulder pain, Constant-Murley score, Chinese version, Reliability

## Abstract

**Background:**

Shoulder pain is a common musculoskeletal disorder in Chinese population, which affects more than 1,3 billion individuals. To the best of our knowledge, there has been no available Chinese-language version of measurements of shoulder pain and disability so far. Moreover, the Constant-Murley score (CMS) questionnaire is a universally recognized patient-reported questionnaire for clinical practice and research. The present study was designed to evaluate a Chinese translational version of CMS and subsequently assess its reliability and validity.

**Methods:**

The Chinese translational version of CMS was formulated by means of forward-backward translation. Meanwhile, a final review was carried out by an expert committee, followed by conducting a test of the pre-final version. Therefore, the reliability and validity of the Chinese translational version of CMS could be assessed using the internal consistency, construct validity, factor analysis, reliability and floor and ceiling effects. Specifically, the reliability was assessed by testing the internal consistency (Cronbach’s α) and test-retest reliability (intraclass coefficient correlation [ICC]), while the construct validity was evaluated via comparison between the Chinese translational version of CMS with visual analog scale (VAS) score and the 36-Item Short Form Health Survey (SF-36, Spearman correlation).

**Results:**

The questionnaire was verified to be acceptable after distribution among 120 subjects with unilateral shoulder pain. Factor analysis had revealed a two-factor and 10-item solution. Moreover, the assessment results indicated that the Chinese translational version of CMS questionnaire harbored good internal consistency (Cronbach’s α = 0.739) and test-retest reliability (ICC = 0.827). In addition, the Chinese translational version of CMS was moderately correlated with VAS score (*r* = 0.497) and SF-36 (*r* = 0.135). No obvious floor and ceiling effects were observed in the Chinese translational version of CMS questionnaire.

**Conclusion:**

Chinese translational version of CMS exhibited good reliability, which is relatively acceptable and is likely to be widely used in this population.

## Background

Shoulder pain is a medical discomfort and socioeconomic problem, commonly seen in both developed and developing countries [1]. Shoulder pain and stiffness can hamper your ability to move freely, leading to difficulties in work and/or daily activities, which have caused a great deal of pain on patients and severe burdens on the society [2–5]. Shoulder complaints are not self-limiting in most cases, as reported that approximately 50% of patients finally consult a general practitioner after 1 year [1]. The aim of treatment for shoulder pain includes recovery of shoulder joints function and alleviation of pain intensity. Questionnaires, which assess pain intensity and dysfunction of shoulder joint, are extensively used in trials on shoulder pain, so as to evaluate the treatment effectiveness.

Therefore, reliable and valid tools to assess shoulder function are important in research and clinical practice [6]. The Constant-Murley score (CMS) questionnaire is one of the most frequently used shoulder-specific scoring system, which can be specifically used to assess pain and disability in patients with shoulder complaints [7]. CMS questionnaire is recognized as an excellent gauge in assessment of response to treatment in patients with subacromial pain. More importantly, the global assessment score can distinguish patients with a slightly better response from those with a much better response [8]. Notable for its sensitivity and reproducibility, CMS questionnaire harbors good inter- and intra-observer reliability [7, 9, 10].

However, appropriate translation and cultural adaptation should be conducted before an evaluation instrument can be used effectively in another culture or language. The CMS questionnaire has recently been translated and cross-culturally adapted to the Danish and Brazilian populations [11, 12]. Shoulder pain is a common musculoskeletal disorder in the Chinese population, affecting more than 1,3 billion individuals. To the best of our knowledge, no Chinese-language measurements of shoulder pain and disability have been accessible so far. This study was thereby conducted, aiming to translate the CMS into Chinese, and subsequently to evaluate its measurement properties in Chinese population based on the published guideline from the American Association of Orthopedic Surgeons (AAOS) Outcome Committee [13].

## Method

### Design

The CMS was translated into Chinese in accordance with the cross-cultural-adaptation guidelines from AAOS Outcome Committee [13].

### Ethical Considerations

The entire protocol was approved by the Ethics Committee of Longhua Hospital. All subjects participating in the study were well informed of the details and provided the written, informed consent.

### Participants, therapists and centers

The study was conducted in four hospitals, including Longhua Hospital and Yueyang Hospital, which were affiliated to Shanghai University of Traditional Chinese Medicine, as well as Jing’an District Central Hospital and Guanghua Hospital. Native Chinese-speaking patients aged 18–70 years suffering from unilateral shoulder pain for at least 1 month were enrolled in the study. Meanwhile, patients were ineligible if they harbored shoulder pain due to other pathologies, such as bone fracture, tumor, tuberculosis, surgical treatment of any condition in the affected shoulder within the past 12 months, or were diagnosed with mental disorders or had insufficient understanding of Chinese characters.

The estimation of sample size was based on a method, developed to calculate the number of subjects required to measure good internal consistency, good construct validity, good reliability, and floor and ceiling effects of the CMS. Moreover, the sample size is required to be up to seven times of the number of items (8 items*7 = 56), meanwhile, it should be more than 100. Therefore, the sample size should be over 100 in the study [14].

### Intervention

#### Instruments

##### Constant-Murley score (CMS)

CMS was classified into four subscales, including pain (15 points at most), activities of daily living (20 points at most), range of motion (40 points at most), and strength (25 points at most). A higher total score suggested higher function of shoulder (range, 0–100) [8].

##### Visual analog scale (VAS) score

VAS score, a simple self-reported method to evaluate pain intensity, was employed in this study. It was a 100-mm horizontal line, with the left endpoint of scale (0 mm) indicating *no pain,* while the right endpoint (100 mm) suggesting *the worst pain imaginable*. The patients were required to select the point on the line that best represented their perception of pain level [15].

##### Short Form Health Survey (SF-36)

Quality of life was evaluated using the 36-item Short-Form Health Survey (SF-36), which included evaluation of physical function, role-physical, bodily pain, general health, vitality, social functioning, role-emotional and mental health. Scores for each dimension ranged from 0 (poor health) to 100 (good health) [16, 17]. These eight scales could be aggregated into two summary parameters, which were, Physical Component Summary score (PCS) and Mental Component Summary score (MCS). To be specific, PCS included physical functioning, role-physical, bodily pain and general health; while MCS was constituted by the scales of vitality, social functioning, role-emotional and mental health [18].

#### Translation process

The cross-cultural adaptation of a scale includes both translation and cultural modification. The cross-cultural adaptation steps, described by Beaton et al. [13], were employed to translate the CMS questionnaire into Chinese version.

##### Stage I

Firstly, the original English version of CMS was translated into Chinese independently by two bilingual native Chinese-speaking translators (T1, T2) with different educational and job profiles. Of them, one translator was an orthopedic doctor, who was well aware of the concepts examined in the questionnaire, while the other one was an economic PhD without any medical background. Moreover, a third translator was designated to review the translations from both translators thoroughly, so as to make cultural and vocabulary adaptations, with the synthesized version being named T1–2.

Next, T1–2 was translated back into English independently by two native English-speaking individuals, whose second language was Chinese. In addition, translational difficulties, cultural diversity, conceptual equivalence, and vocabulary differences were highlighted using such translation technique [19]. Notably, the back-translators were not aware or informed of the concepts explored, and had never seen the original version of the questionnaire, either. One of them held a PhD in economics and the other one was a professional translator. Thus, neither of them had a medical background. The back-translators were inaccessible to the original English questionnaire during the entire translation process. Subsequently, the back-translated version was compared with the original version by an expert committee.

Specifically, all Chinese and English translations were then reviewed by a bilingual expert committee, which was constituted by all translators, authors, an orthopedic surgeon, a rehabilitation physician, a methodologist, and a linguist. The semantic, idiomatic, and conceptual equivalences of the items and answers had been deeply explored by the committee, so as to identify all difficulties or mistakes. The Chinese version of the CMS questionnaire was accepted only after the committee had reached consensus on all discrepancies.

Finally, the pre-final version of the CMS questionnaire was distributed to 35 patients who met the inclusion criteria. In this way, the problem of whether all questions were clear and comprehensible could be determined. Besides, all findings were reevaluated by the expert committee.

##### Stage II: testing the final version

A booklet containing all questionnaires (final version of CMS questionnaire, VAS for shoulder pain, SF-36, participant information, and informed consent) was used in the present study. The booklet was distributed to over 100 patients, who met the inclusion criteria, for a validity test of the final version. However, only 50 of them had completed the test as requested on time. Therefore, these patients were asked to complete the same questionnaire 7 days later as a retest. Demographic characteristics and other related history of all patients were recorded.

### Data analysis

Exploratory factor analysis was originally performed, and the number of extracted factors was determined using the principal-component analysis (PCA) [20]. Promax rotation was applied, meanwhile, items with a factor loading of over 0.40 were included in the factor [21]. Internal consistency was evaluated by calculating the Cronbach’s α. In general, α > 0.7 was regarded as acceptable; however, it should not be higher than 0.95, in order to avoid redundancy [14, 22, 23].

Reliability was investigated through checking the internal consistency and test-retest reliability. CMS questionnaires received within 7 days after the baseline test were tested using the intraclass correlation coefficient (ICC). ICC ranged from 0 to 1, with a higher value indicating greater repeatability. More importantly, an ICC of >0.75 suggested good reliability, while an ICC of <0.4 stood for poor reliability [24].

Criterion-related validity was determined using the concurrent-validity method by means of evaluating the relationships between the CMS and VAS, as well as between the CMS and the Chinese version of the SF-36 [17].

In the study, the floor and ceiling effects were evaluated in the general results of the CMS. Ceiling and floor effects were considered to be present if more than 15% of respondents achieved the lowest (0) or highest (100) possible total score.

## Results

### Flow of participants, therapists, centers throughout the study

#### Translation and cross-cultural adaptation

The final Chinese version of CMS questionnaire was shown in Supplemental Material 1, while the original English version was available online [7]. Several points were modified to adjust the CMS into the Chinese language and culture.

Furthermore, we also consulted Dr. Constant for more details on rating the activities of daily living and work, recreation/sport, and sleep. With regard to sleep, a patient would be scored 2 points if normal sleep was not greatly interfered by shoulder pain. Moreover, other factors that might affect sleep but were not involved the shoulder joint, were not taken into account. A patient would be scored 1 point if he/she was awakened by shoulder pain but was able to return to sleep by repositioning or taking a sleeping tablet. The score of 0 was rated for a patient who was awakened or had difficulty in getting sleep due to the shoulder pain, such that they could not get a reasonable night’s sleep.

#### Sample Description

**Fig. 1:**
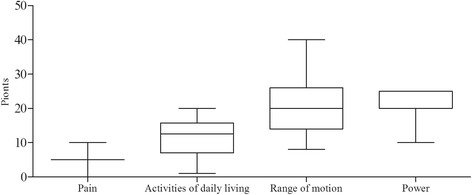
The range of the CMS subsections

35 patients (including 20 females, and 15 males) were tested in the pre-final pilot study. Notably, 120 participants (69 females, 51 males) were involved in the final test. Female patients (57.50%) with 55.64 ± 9.49 years old were associated with higher incidence of shoulder pain. The shoulder pain lasted for an average of 62.75 ± 15.96 weeks. Demographic and clinical characteristics of patients were listed in Table 1, while the range of CMS sections was shown in Fig. 1.

**Table 1:** The demographic characteristics of the participants at two stages

Characteristic		Prefinal group (*n* = 35)	Validity group (*n* = 120)
Age, mean (S.D.), years		59.94 ± 9.21	55.64 ± 9.49
Age distribution	18–30	3	15
	30–50	15	24
	50–70	18	81
Gender	Male	15	51
	Female	20	69
Disease	Scapulohumeral periarthritis	18	68
	Rotator cuff injury	9	31
	Bicipital tendinitis	3	6
	Other	5	15
Disease duration, mean (S.D.),weeks		8.81 ± 9.20	27.98 ± 51.39
Side	Left	16	55
	Right	19	65
Stage	Acute (<6 wk)	3	32
	Sub-acute (6 wk.–3 mo)	9	30
	Chronic (>3 mo)	23	58
VAS score		/	62.75 ± 15.96
SF-36		/	58.88 ± 14.32
Constant-Murley score		57.46 ± 17.80	47.78 ± 15.34
Occupation	Not working	20	63
	Physically light work	7	22
	Physically medium work	3	23
	Physically heavy work	5	12

Data expressed as mean ± standard deviation or n. *SF-36* 36-Item Short Form Health Survey, *VAS* visual analogue scale

Factor analysis of the CMS questionnaire was performed with the promax rotation, meanwhile, a two-factor structure was extracted by PCA. Factor 1 included items of pain, forward elevation, lateral elevation, external rotation, internal rotation and power from the CMS questionnaire. Factor 2 contained items of work, recreation/sport, sleep and positioning from the CMS questionnaire. Loadings of all items were presented in Table 2.

**Table 2:** Explanatory factor analysis with promax-rotated factor loadings of the Constant-Murley Score items

Constant-Murley score item	Score of each item	Principal Component Coefficients ≥0.4	
		Factor 1	Factor 2
Pain	5.24 ± 2.86	0.569	
Activities of daily living	11.32 ± 6.00		
Work	1.84 ± 1.12		0.766
Recreation/sport	1.67 ± 1.12		0.794
Sleep	0.88 ± 0.67		0.498
Positioning	6.14 ± 3.72		0.645
Range of motion	20.22 ± 8.20		
Forward elevation	5.96 ± 2.52	0.766	
Lateral elevation	5.82 ± 2.21	0.848	
External rotation	3.67 ± 2.84	0.407	
Internal rotation	4.99 ± 2.32	0.753	
Power	22.57 ± 1.13	0.649	

##### Internal consistency

Cronbach’s α was 0.739 for the CMS questionnaire, indicating a high degree of internal consistency.

##### Reliability

Data distributions at test and retest, as well as the reliability of CMS questionnaire were shown in Table 3. A total of 58 patients completed the questionnaires twice at an interval of 7.01 ± 1.05 days. The CMS was slightly higher in the retest (mean score = 4.11) than in the first test. The ICC was 0.827, suggesting an excellent test-retest reliability.

**Table 3:** Results of test-retest analyses for the Constant-Murley score

	Mean ± SD
Test Constant-Murley score	42.75 ± 12.97
Retest Constant-Murley score	46.86 ± 12.71
ICC	0.827

*ICC* intraclass correlation coefficient, *SD* standard deviation

##### Validity

120 patients with shoulder complaints were enrolled as the participants in the survey. Correlations of CMS with VAS and SF-36 subscales scores were displayed in Table 4. The results suggested moderate to low correlations of CMS with VAS score (*r* = 0.497) and SF-36 (*r* = 0.135).

**Table 4:** Correlation (r) for the SF-36 and VAS with the Chinese version of the Constant-Murley score

	Pearson correlation with Constant-Murley score
VAS	0.497
SF-36	0.135
SF-PCS	0.418
SF-MCS	0.198

*SF-36* 36-item Short-Form Health Survey, *SF-MCS* SF-36 mental component summary, *SF-PCS* SF-36 physical component summary, *VAS* visual analog scale

##### Floor and ceiling effect

No floor or ceiling effects were found in this study, and none of the patients had reported the worst or best possible CMS.

## Discussion

In the study, we demonstrated that the Chinese version of CMS questionnaire was a valid, reliable and internally consistent instrument for assessing patients with shoulder complaints, which displayed no floor or ceiling effects. All items in CMS questionnaire had the loadings of >0.40 after two-factor PCA examination. Furthermore, the Cronbach’s α of 0.739 indicated good internal consistency. The test-retest results (ICC = 0.827) confirmed excellent reliability. In addition, it was revealed that CMS correlated well with VAS score (*r* = 0.497); however, poor correlation of CMS and SF-36 scores was observed in the study (*r* = 0.135). Taken together, these results demonstrated that the CMS questionnaire was useful in evaluating Chinese patients with shoulder pain in both clinical practice and research settings.

The translation procedure was completed in strict accordance with the guidelines from the AAOS Outcome Committee [13]. Some modifications were implemented in the translation, aiming to increase the specificity of CMS questionnaire in detecting shoulder pain in the Chinese population. Determination of power was the major difference between English and Chinese versions of CMS questionnaires. In the past, the score given for normal strength was 25 since a healthy man could resist 25 lb. Nonetheless, it is now recommended that strength be measured using a dynamometer in 90° of lateral elevation (shoulder abduction) in the scapular plane in the presence of wrist pronation [7]. Thereby, patients who are unable to achieve the test position of 90° are assigned a score of zero [25]. This may be the ideal measurement of power; however, it is not practical in clinical applications. Consequently, the traditional muscle classification was applied in the Chinese version of CMS questionnaire.

In our study, test validities, namely, correlations of CMS with VAS score and SF-36 subscales, were measured. Correlation values of or over 0.40 were considered as satisfactory (0.81–1.0 excellent, 0.61–0.80 very good, 0.41–0.60 good, 0.21–0.40 fair, and 0–0.20 poor) [22]. CMS exhibited good correlation with VAS score, while poor correlation of CMS with SF-36 subscales was seen. In line with a previous study, it was speculated that the lower-extremity function was involved in various SF-36 items, thus, a lower response in shoulder-specific function was achieved, compared with that in CMS [8]. In addition, the Chinese parameter of SF-36 was a global subjective outcome measurement of daily living, and was further aggregated into two summary measures, including physical and mental measures. Of note, CMS has focused more solely on the physical function of the shoulder rather than the mental function.

This CMS questionnaire has been translated into Danish and Brazilian [11, 12]. Validation of the Danish version was conducted on 45 patients with the age of 59.0 ± 17.7 years old, in which agreement, floor and ceiling effects, and intra- and inter-rater reliability were analyzed. However, that study was not adequately powered to determine good agreement, floor and ceiling effect, and good intra- and inter-rater reliability due to the relatively small sample size [11]. 120 participants were enrolled in our study, in which, more analyses of cross-cultural adaptation were carried out. Compared with the study by Moeller et al., the present study has provided more reliable results for the cross-cultural adaptation of the CMS. The Brazilian adaptation has enrolled 110 subjects, which indicated a strong negative correlation between CMS and DASH score [12].

The study harbored the following advantages. Firstly, participants were recruited from four different hospitals, which could better reflect the majority and diversity of the population. Secondly, the translation procedure was completed according to the guidelines established by the AAOS Outcome Committee, leading to more explicit and reliable results. However, there existed several limitations in the present study. Firstly, our inclusion criteria were limited to patients who suffered from unilateral shoulder pain (such as scapulohumeral periarthritis and degenerative changes) for 1 month, which may limit the applicability of the findings to other shoulder conditions. Secondly, sensitivity analysis of the CMS was not carried out, which may have involved more factors, such as the effectiveness of the intervention as well as the type and severity of disease. Moreover, a more comprehensive project may be necessitated. In addition, no comparison with other shoulder questionnaires was conducted in this study to test the convergent validity, probably due to the absence of Chinese-language measurements of shoulder pain and disability. Therefore, a prospective study that including sensitivity analysis and covering more shoulder conditions is recommended.

## Conclusions

In conclusion, our study revealed that the Chinese version of CMS harbored good internal consistency and excellent test–retest reliability. Moreover, our findings suggested that the Chinese version of CMS questionnaire was a reliable and valid instrument for evaluating shoulder pain in the Chinese population, which deserved wide application in patients with shoulder pain in both clinical practice and research settings. However, further prospective studies enrolling more shoulder diseases are warranted to complete the sensitive analysis and make the outcomes more convincing.

## Abbreviations

AAOS: American Association of Orthopedic Surgeons
CMS: Constant-Murley score
ICC: Intraclass correlation coefficient
PCA: Principal-component analysis
SF-36: Short Form Health Survey
VAS: Visual analog scale
